# Epithelial-to-mesenchymal transition, circulating tumor cells and cancer metastasis: Mechanisms and clinical applications

**DOI:** 10.18632/oncotarget.18277

**Published:** 2017-05-26

**Authors:** Xiao-Xiang Jie, Xiao-Yan Zhang, Cong-Jian Xu

**Affiliations:** ^1^ Obstetrics and Gynecology Hospital, Fudan University, Shanghai 200011, People's Republic of China; ^2^ Department of Obstetrics and Gynecology of Shanghai Medical School, Fudan University, Shanghai 200032, People's Republic of China; ^3^ Shanghai Key Laboratory of Female Reproductive Endocrine Related Diseases, Shanghai 200011, People's Republic of China; ^4^ Institute of Biomedical Sciences, Fudan University, Shanghai 200032, People's Republic of China

**Keywords:** epithelial-to-mesenchymal transition, circulating tumor cells, metastasis, EMT markers

## Abstract

Epithelial-to-mesenchymal transition (EMT) endows epithelial cells with enhanced motility and invasiveness, allowing them to participate in many physiological and pathological processes. Epithelial-to-mesenchymal transition contributes to the generation of circulating tumor cells (CTCs) in epithelial cancers because it increases tumor cell invasiveness, promotes tumor cell intravasation and ensures tumor cell survival in the peripheral system. Although the contribution of epithelial-to-mesenchymal transition to tumor cell invasiveness has been confirmed, the role epithelial-to-mesenchymal transition plays in metastasis remains debated. As a favorable material for a “liquid biopsy”, circulating tumor cells have been shown to have promising values in the clinical management of tumors. Furthermore, an increasing number of studies have begun to explore the value of CTC-related biomarkers, and some studies have found that the expression of EMT and stemness markers in circulating tumor cells, in addition to CTC detection, can provide more information on tumor diagnosis, treatment, prognosis and research.

## INTRODUCTION

Epithelial cancer metastasis is a multi-step process that includes a loss of intercellular connections, the invasion of basal membrane and surrounding tissues, intravasation into venous or lymphatic vessels that generates circulating tumor cells (CTCs), survival in the peripheral system, extravasation and proliferation at secondary sites. In this metastatic cascade, epithelial-to-mesenchymal transition (EMT) is believed to play an important role [1–3]. EMT is characterized by decreased epithelial properties and increased mesenchymal attributes and has been implicated in a number of physiological processes, such as embryonic development and pathological conditions, including organ fibrosis and cancer progression [2–4].

One important aspect of EMT's role in cancer is that EMT contributes to the generation of CTCs. CTCs are tumor cells released into blood and/or lymphatic vessels that can circulate in the human body, which are predestined sources of metastasis as the “seeds” in Paget's “seed and soil” hypothesis [5]. Moreover, EMT has been shown to contribute to tumor resistance [6–8] and may also be related to tumor cell stemness [9, 10]. However, because of technical restrictions and a lack of convincing *in vivo* evidence, the role of EMT in cancer metastasis remains debated. Some believe that EMT is of great importance in the formation of metastases, whereas others hold that the effect of EMT might have been overestimated, given the lack of convincing evidence. In fact, some have suggested that EMT is not required for metastasis [6, 8, 11–13].

Because of their crucial role in metastasis, CTCs have had attracted much attention since their discovery. Furthermore, the development of CTC detection technology has facilitated the real-time dynamic monitoring of cancer by using CTCs as a material for “liquid biopsies”, which are superior to traditional biopsy [14, 15]. In the last decade, the detection and enumeration of CTCs have been shown to provide information on prognosis, metastasis, therapeutic efficacy and chemoresistance in several cancer types [16–20]. Recent studies of CTCs are not limited to the detection and enumeration of these cells, and increasing efforts have been made to elucidate the molecular features of CTCs and the potential values of these biomarkers, among which EMT markers are of great interest.

In this review, we summarize the mechanisms underlying EMT and their role in CTC generation, shed light on the controversial role of EMT in metastasis, and review recent advances that have been made in the clinical application of EMT markers in CTC detection.

## THE MECHANISMS UNDERLYING EMT AND THEIR ROLE IN METASTASIS

### The mechanisms underlying EMT

Epithelial-to-mesenchymal transition (EMT) is a multi-step process involving many molecular and cellular changes, including the down-regulation of epithelial proteins such as E-cadherin, claudins and cytokeratins and the up-regulation of mesenchymal proteins, such as N-cadherin, fibronectin and vimentin, which endow the cell with increased motility and invasiveness [1–3, 21].

**Figure 1:**
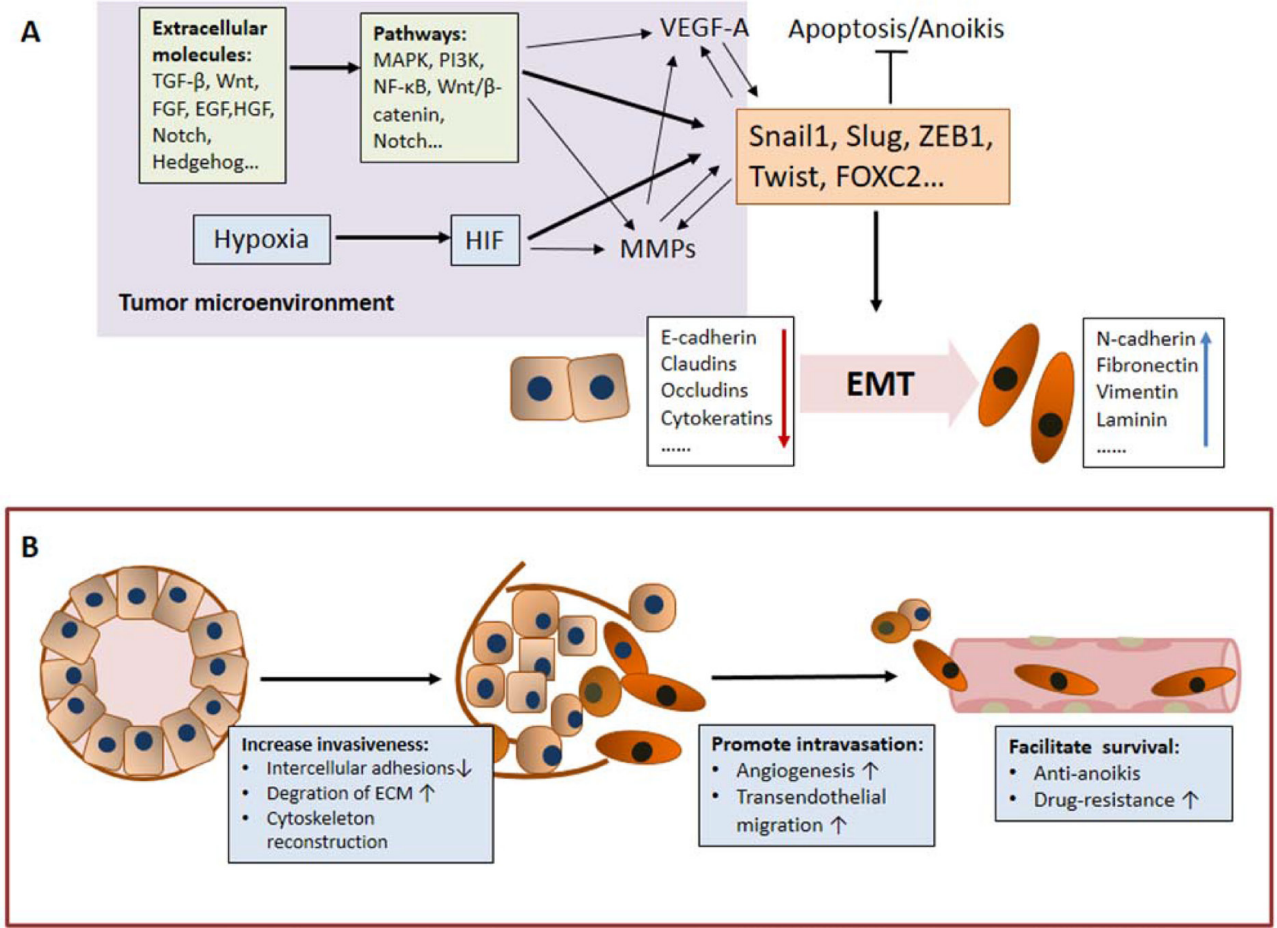
The mechanisms underlying the role of EMT in CTC generation (**A**) The EMT-related regulatory network. EMT-inducing transcription factors (EMT-TFs), including Snail 1, Snail 2 (Slug), ZEB1, and Twist, play a central role in this network and regulate molecular changes during EMT. Some important extracellular molecules in the tumor microenvironment, such as TGFβ, HGF, FGF, Wnt and Notch, bind to their respective receptors to induce EMT and are consequently also important components in the EMT regulatory network. Hypoxia, a significant aspect in cancer progression, triggers EMT and participates in the EMT regulatory network. Notably, the EMT regulatory network is an interactive, integrated and precisely regulated network that is involved in the generation of CTCs. (**B**) EMT promotes CTC generation by increasing tumor cell invasiveness, promoting tumor cell intravasation and facilitating tumor cell survival in the peripheral system.

These molecular changes during EMT are regulated by transcription factors called EMT-inducing transcription factors (EMT-TFs), including Snail 1, Snail 2 (Slug), ZEB1, Twist, TCF4, and FOXC2 [22, 23]. In addition to EMT-TFs, some extracellular molecules in the tumor microenvironment (TGF-β, FGF, EGF, HGF, Wnt, Notch, Hedgehog, etc.) and related pathways (MAPK, PI3K, NF-κB, Wnt/β-catenin, Notch, etc.) are thought to induce EMT [23–27]. Moreover, hypoxia results in the accumulation of hypoxia-inducible factor (HIF), and HIF-1α activates EMT-TFs, such as Twist and Snail, to induce EMT [28, 29] (Figure 1).

EMT plays a role in a number of physiological processes and pathological conditions. EMT has been categorized into three types based on function [2]: type I is involved in embryonic development, type II participates in wound healing and fibrosis, and type III is associated with cancer progression, which is the focus of this review.

### EMT promotes CTC generation

The generation of CTCs includes several steps: the detachment from the tumor mass, invasion of the basal membrane and surrounding tissues, entry of vessels and survival in the peripheral system. Specifically, EMT and related regulatory networks primarily promote CTC generation in three aspects: i) increase tumor cell invasiveness, ii) promote tumor cell intravasation and iii) facilitate tumor cell survival in the peripheral system. (Figure 1)

As shown in Figure 1, the EMT regulatory networks involves not only molecular changes directly regulated by EMT-TFs but also other related factors and pathways, such as HIF, TGF-β and VEGF-A, which together promote the ultimate generation of CTCs.

Epithelial cells are immobile due to the precise regulation of strong cell-cell and cell-extracellular matrix adhesions, which consist of adherent junctions, tight junctions and desmosomes, and the well-constructed cytoskeleton. During EMT, the key components of intercellular junctions, such as E-cadherin, claudins, occludins and desmosomes, are directly down-regulated by EMT-TFs, such as Snail, Slug and SIP1 [22, 30–32]. Some cell and animal assays also demonstrated that the reorganization of adhesive molecules is associated with increased invasiveness [33–35]. The phenotype switch from epithelial to mesenchymal during EMT also involves the reconstruction of the cytoskeleton, which changes their morphology to a spindle-like shape that is appropriate for migration [36]. In addition to adhesive molecules, EMT-TFs can also induce the expression of matrix metalloproteinases (MMPs), which facilitate the degradation of the basal membrane and surrounding tissues [37–39]. Remarkably, some important extracellular factors, such as TGF-β, FGF and Wnt, participate in the regulatory network by inducing EMT and/or the expression of MMPs [23–27, 29, 40, 41]. The mechanisms of hypoxia in this regulatory network are similar to those of extracellular factors, as shown in Figure 1 [28, 29, 42, 43].

The EMT regulatory network also promotes angiogenesis and facilitates cancer cell intravasation. Specifically, EMT-TFs such as Snail and Slug, can promote blood vessel formation by inducing the expression of vascular endothelial growth factor A (VEGF-A) in subcutaneous xenograft tumor models [44–46]. Other factors in the regulatory network, such as Notch and HGF, can also promote angiogenesis via a similar mechanism [47–49]. Remarkably, VEGF and HIF1-α were also expressed on CTCs [50]. Moreover, EMT-induced and TGF-β-induced proteases, especially MMPs, can also promote angiogenesis and intravasation [51, 52]. Moreover, newly formed, tumor-associated vessels are often malformed and leaky, which facilitates tumor cell invasion [53]. Specifically, EMT-TFs or EMT-related factors have been shown to enhance transendothelial migration, which further supports that EMT promotes tumor cell intravasation [54, 55].

CTCs do not easily survive in the peripheral system because they may encounter strong anoikis signals and chemotherapy or radiotherapy. Nevertheless, EMT can facilitate tumor cell survival in the peripheral system by allowing cancer cells to avoid apoptosis, anoikis, and senescence and promote drug resistance [56, 57]. EMT-TFs, such as Snail, Slug, Twist and SIP1, can protect CTCs from anoikis by disturbing normal apoptotic cascades, resisting senescence and/or cooperating with TrkB [58–62]. EMT-TFs can reportedly endow tumor cells with resistance to chemotherapy and radiotherapy in several cancer types [6, 8, 63]. For example, Snail and Slug directly contribute to cisplatin resistance in ovarian cancer [64, 65]. Moreover, the inhibition of EMT can restore the chemosensitivity, indicating that the EMT-TFs may be a potential target for the treatment of therapy resistance [7, 66, 67].

Remarkably, many regulatory loops exist between the components of the EMT regulatory network [43, 45, 68–71], as shown in Figure 1. For example, EMT-TFs can induce the expression of MMPs, whereas some proteases can reverse EMT [69, 70]. VEFG-A plays a similar role in EMT [45, 71]. In summary, the EMT-related regulatory network is an interactive, integrated and precisely regulated network due to the association between EMT, extracellular factors (TGF-β, FGF, Notch, etc.) and hypoxia, which are all important aspects in cancer progression. As the central part of the regulation network, EMT, accompanied by the related factors and pathways, plays an important role in the generation of CTCs by promoting cell invasion, angiogenesis, intravasation, therapy resistance and survival.

### EMT's role in metastasis

**Figure 2:**
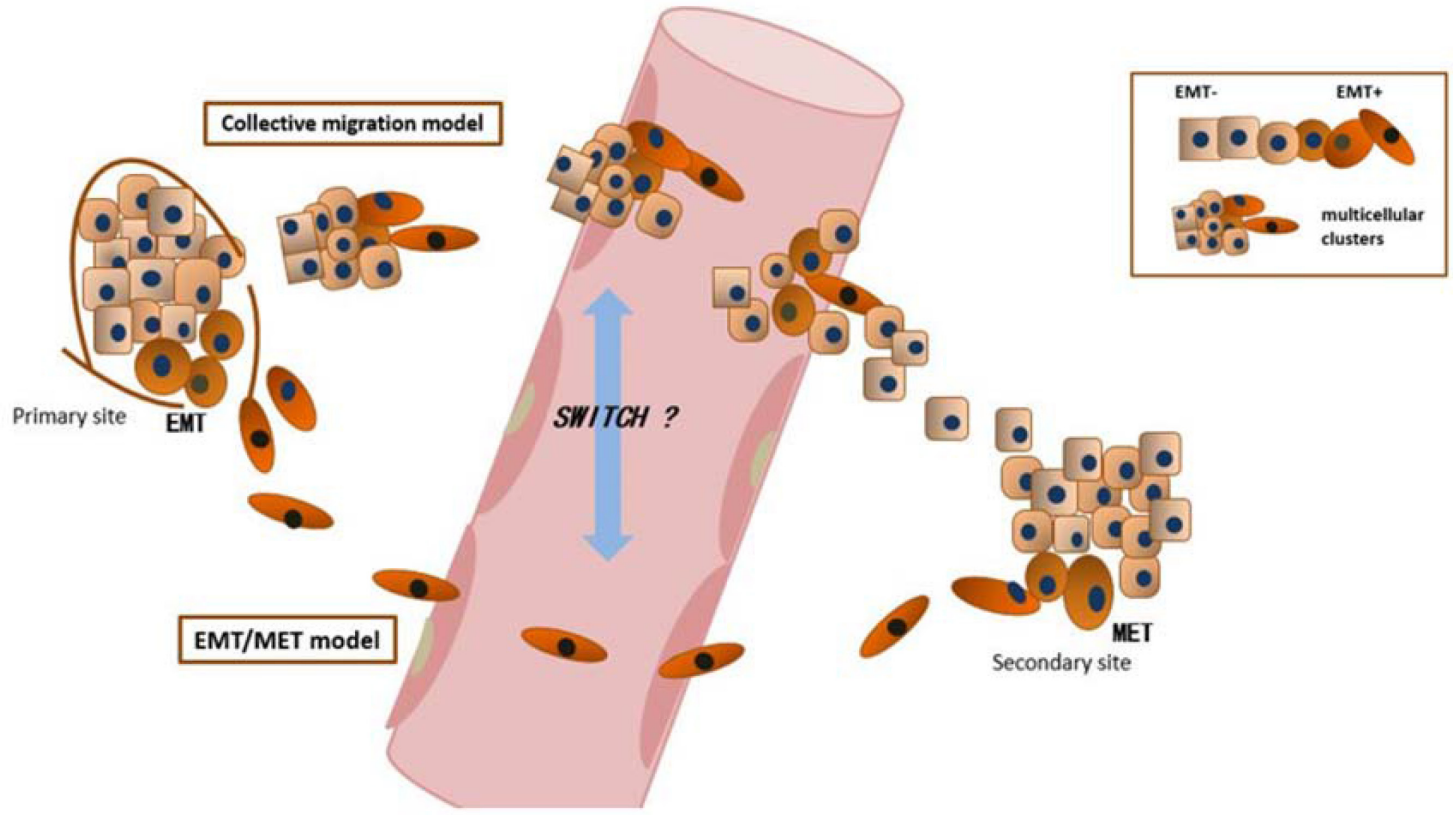
EMT and metastatic models EMT/MET model: epithelial cancer cells must first undergo EMT to become invasive and motile and generate CTCs; CTCs circulate around the body and extravasate to distant sites; after extravasation to secondary sites, cancer cells must undergo the reverse process of EMT, MET (mesenchymal-to-epithelial transition) to restore epithelial properties, allowing them to ultimately colonize distant sites and form metastases. Collective migration model: instead of migrating as a single cell, cancer cells that have undergone various degrees of EMT coexist as multicellular clusters and migrate collectively, with the more motile invasive mesenchymal-like cells aggregating at the invasive front of multicellular clusters to “pave” the way, whereas epithelial-like cells retain their epithelial properties, follow behind and seize the opportunity to proliferate and colonize at distant sites after extravasation. Notably, the EMT/MET model and collective migration model may be not independent or diametrically opposed. Tumor cells may switch between the two mechanisms under certain circumstances, or the two mechanisms may synergistically effect metastases.

The contribution of EMT to tumor cell invasiveness and CTC generation has been confirmed, but its role in the metastatic cascade remains debated. Two metastatic models involving EMT have been proposed (Figure 2). The first and most widely known model proposes that cancer cells must first undergo EMT to become invasive and generate CTCs and then undergo the reverse of EMT, MET (mesenchymal-to-epithelial transitions), to restore epithelial properties after extravasation into secondary sites and facilitate metastatic growth [72–75]. This process of EMT and subsequent MET is very common and important during embryonic development and some postnatal gland development. Thus, this process can be expected to play a role in cancer progression [73, 76]. Specifically, this EMT/MET metastatic model can explain the fact that the histopathological features of secondary tumor sites resemble those of the primary site [73]. Furthermore, the switch between EMT and MET and phenotypic plasticity have been reported in some cancer types [77, 78]. For example, Chao et al. have reported that E-cadherin was re-expressed due to MET and may play a role in promoting cell survival at metastatic sites [79, 80]. Although clinical studies have indicated that MET occurs in distant sites, a lack of *in vivo* evidence of MET at distant sites reduces the credibility of this model. In recent years, increasing evidence from both *in vitro* and *in vivo* experiments has supported this metastatic model. For example, Chaffer et al. used a series of bladder cancer cell lines to study the role MET in the metastasis cascade and found that reversion to epithelial characteristics in a mesenchymal-like cancer cell line, i.e., regaining an epithelial phenotype, was favorable in the latter stages of the metastatic cascade; this finding confirmed the suspected role of MET in secondary cancer growth [81, 82]. Moreover, Banyard, J and colleagues used an *in vivo* cycling strategy to select metastatic cancer cells from the lymph nodes of mice bearing orthotopic DU145 human prostate tumors and observed a shift to an epithelial phenotype in progressive lymphatic cancer cells, providing evidence for spontaneous MET *in vivo* [83]. Ocana, O and colleagues induced MET by silencing Prrx1, an EMT inducer, and found that the loss of Prrx1 was required for migratory cells to colonize a secondary organ *in vivo* [84]. Tsai, JH et al. came to a similar conclusion: they found that turning off Twist 1, an important EMT-TF, to allow the reversion of EMT was essential for cancer cells to form metastases in distant sites [85].

However, the classic EMT/MET model has been challenged in recent years [11–13]. Although the importance of EMT in endowing cancer cell with enhanced invasiveness and motility has been confirmed, the role of MET in the metastatic cascade remains debated. Increasing evidence suggests that MET plays a role in the latter stages of the metastatic cascade, but some researchers question the necessity of MET to complete colonization at distant sites. Based on the concept of “collective” or “cohort” migration [86], they introduced the second metastatic model discussed in this review: epithelial-like and mesenchymal-like cancer cells can cooperate with each other during collective migration to achieve metastasis instead of undergoing the difficult process of EMT and subsequent MET [86, 87]. EMT is not an on/off switch, and the phenotypic transition from epithelial to mesenchymal in cancer cells consequently would give rise to many hybrid phenotypes that possess the properties of both cell types [88]; this phenotype plasticity facilitates collective migration. The collective migration model hypothesizes that many hybrid phenotypes coexist as multicellular clusters and migrate collectively, with the more motile invasive mesenchymal-like cells aggregating at the invasive front of multicellular clusters to “pave” the way, whereas epithelial-like cells follow behind and seize the opportunity to proliferate and colonize distant sites after extravasation [87]. Accordingly, multicellular cancer fragments, so-called tumor micro-emboli or CTC clusters, have long been observed in the clinic and correlate with distant metastasis [13, 89]. Moreover, several *in vivo* studies support this model. For example, Tsuji and colleagues established an animal model by subcutaneously and intravenously injecting EMT-derived cells and non-EMT cells, respectively, and found that EMT-derived cells were able to invade the blood stream but failed to establish lung metastases, whereas non-EMT cells could not invade but were able to establish lung metastases when injected intravenously. Surprisingly, lung metastases formed when a mixture of EMT-derived and non-EMT cells were co-inoculated subcutaneously, suggesting cooperation between epithelial and mesenchymal cancer cells during metastasis [90, 91]. Banyard, J and colleagues also reported that the epithelial-like DU145-LN4 cell line invaded in a collective migration pattern into lymphatic vessels in their model [92]. Fischer, KR et al. established an *in vivo* EMT lineage tracing system to monitor EMT in a triple-transgenic mouse model, in which breast-to-lung metastasis occurred spontaneously and EMT was “visible”, if it occurred. Notably, lung metastases mainly consisted of non-EMT cancer cells, which maintained epithelial phenotype during metastasis in their study, contradicting the original EMT/MET hypothesis [6]. Surprisingly, when they inhibited EMT by overexpressing miR-200, lung metastases were not affected, leading them to conclude that EMT was not required for lung metastasis [6]. Zheng, XF drew a similar conclusion: the suppression of EMT by deleting Snail or Twist in genetically engineered mouse models of pancreatic ductal adenocarcinoma (PDAC) did not affect the development of metastases [8], which also suggested that EMT does not play a role in metastasis. However, the contribution of EMT to the metastatic cascade should not yet be discounted because EMT is complex and precisely controlled. Therefore, the effects of EMT *in vivo* cannot be easily and completely eliminated. And the role of partial EMT may have been underestimated in these aforementioned models.

Notably, although the classic EMT/MET model and collective migration model are conflicting, these two models both state that the mesenchymal phenotype is more invasive and motile than the epithelial phenotype and that the epithelial phenotype favors proliferation compared with the mesenchymal phenotype. Thus, the assumption that the tumor takes advantage of the mesenchymal phenotype in the early stage of the metastatic cascade, when invasion is the main concern, and then exploits the proliferative epithelial phenotype to complete colonization is reasonable. The classic EMT/MET model hypothesizes that cancer cells must undergo this phenotype transition twice, i.e., EMT and subsequent MET, to ensure the optimal phenotype in each stage of the metastatic cascade. Thus, EMT plays a central role in metastasis. Conversely, collective migration model cleverly circumvents these troublesome phenotype transitions and proposes that epithelial and mesenchymal phenotypes cooperate to accomplish metastases. Thus, EMT assists the metastatic cascade in this model. In fact, Chaffer et al., who support the EMT/MET model [82], and Tsuji et al., who back the collective migration model [90], reported similar but slightly different results, which has led to disagreement. Currently, identifying the correct model may be difficult because both are supported by an increasing body of evidence. Specifically, both single-cell motility and collective migration are observed in the circulation. Interestingly, Giampieri, S et al. found that TGFβ-1 acted as a switch between the cohesive phenotype and single cell motility via a transcriptional program, and collective migration seems more practical in lymphatic metastasis than blood-borne metastasis [93]; however, this finding warrants further confirmation. Banyard, J also hypothesized that EMT may not be essential during lymphatic metastasis [75]. Thus, the EMT/MET model and/or collective migration model may not be independent or diametrically opposed; the two models may co-exist and synergistically facilitate the metastatic cascade. Tumor cells may switch between the two mechanisms under certain circumstances or prefer a mechanism for a specific type of metastasis to maximize their colonization of distant sites.

## CLINICAL APPLICATIONS OF EMT MARKERS IN CTCS DETECTION

Circulating tumor cells are highly heterogeneous, and the molecular features of CTC often differ by subpopulation [94–96]; thus, these subpopulations may play different roles in cancer progression. A better understanding of CTC heterogeneity has demonstrated that the molecular features of different CTC subpopulations need to be studied to identify the “evil” CTC subpopulations that are responsible for lethal cancer progression. Therefore, an increasing number of studies no longer focus on simple CTC enumeration and have begun to explore the genotypic and phenotypic characterization of CTCs. For example, researchers have investigated the value of HER 2 expression on CTCs in patients with breast cancer [97, 98], AR gene status of CTCs in prostate cancer [99], EGFR mutations in lung cancer and KRAS mutations in colorectal cancer [100, 101]. Besides these cancer type specific biomarkers, EMT markers can be promising biomarkers of CTCs in different cancers. EMT can endow cells with enhanced invasiveness and motility, drug-resistance, and even stemness properties [2, 30], the CTC subpopulations positive for EMT markers may be responsible for cancer progression [76, 96]. Thus, delineating the expression of EMT markers in CTCs is of great clinical interest.

EMT is believed to generate CTCs with stem cell properties [9], and some studies reported that the overexpression of EMT markers in CTCs is often accompanied by the expression of stem cell markers, such as ALDH 1 and CD133 [102–104]. Below, we review the expression of EMT markers in CTCs and focus on their clinical applications while also examining stem cell markers (Supplementary Table 1).

### EMT markers

The molecular changes during EMT are characterized by the down-regulation of epithelial proteins and up-regulation of mesenchymal proteins, and these changes are regulated by EMT-TFs and related pathways, as stated above. Thus, we can categorize the EMT markers into three types: i) epithelial makers, ii) mesenchymal markers and iii) regulators (Table 1).

**Table 1:** The categorization of EMT markers

Categorization	Marker	Features
**Epithelial markers**	EpCAM	Down-regulated during EMT.
	E-cadherin	Often used to detect CTCs.
	Cytokeratins (CK)	
	Zonula occludins (ZO)	
**Mesenchymal markers**	N-cadherin	Highly expressed in mesenchymal cells. The switch from E-cadherin to N-cadherin is a hallmark of EMT.
	Vimentin	Highly expressed in mesenchymal cells, induces mesenchymal morphology.
	Fibronectin	Regulates cell shape.
**Regulators**	Twist 1	bHLH factor, represses E-cadherin expression.
	Snail 1	Zinc-finger protein, directly represses E-cadherin expression.
	Snail 2 (Slug)	
	ZEB 1/ZEB 2	Zinc-finger E-box-binding homeobox protein, transcriptional repressor.
	Akt and PI3K	The PI3K/AKT/m TOR pathway is associated with proliferation and EMT.
	FoxC 2	Transcriptional activator, induces EMT.

Epithelial markers are molecular biomarkers that are highly expressed in epithelial cells but not expressed or expressed at low levels in mesenchymal cells, such as EpCAM, E-cadherin, cytoketatins, and Zonula occludins (ZO). Epithelial markers are often used to detect CTCs and ensure their epithelial origin. Epithelial cellular adhesion molecule (EpCAM) is an adhesive molecule between epithelial cells and believed to be involved in epithelial malignancies [105]. EpCAM has long been widely used to detect and enumerate CTCs [106, 107], but EpCAM expression is down-regulated if the cancer cells have undergone EMT because it is an epithelial marker. Thus, the use of EpCAM for CTC detection is likely to exclude CTCs that have undergone EMT [108]. Cytokeratins (CKs) are a group of intermediate filaments that comprise the cytoskeleton and have also been used to detect CTCs. Like EpCAM, CKs are down-regulated during EMT and may be not appropriate for CTC detection when CTCs have undergone EMT [108]. Moreover, E-cadherin is an essential component of adherent junctions, and ZOs are proteins of tight junction, which are both involved in the maintenance of epithelial intercellular adhesions and are down-regulated during EMT [109, 110].

Although mesenchymal markers, such as N-cadherin, vimentin, and fibronectin, are highly expressed in mesenchymal cells expressed at low levels in epithelial cells, they are up-regulated during EMT, and the expression of mesenchymal markers by CTC can reflect their EMT status. N-cadherin and E-cadherin both belong to the cadherin family. During EMT, N-cadherin is overexpressed, whereas E-cadherin is down-regulated—this switch from E-cadherin to N-cadherin is a hallmark of CTCs that have undergone EMT [111]. Vimentin is a component of intermediate filaments whose expression facilitates the reconstruction of the cancer cell cytoskeleton during EMT and endows the cancer cell with a spindle-like shape suitable for migration [112, 113].

EMT-TFs and their related pathways regulate the molecular changes during EMT, and these elements themselves can also be markers of EMT, such as Twist, Snail, ZEB and Akt/PI3K. Twist 1 is a transcription factor that acts on the E-box of E-cadherin to down-regulate the expression of E-cadherin, which is associated with cancer progression and a useful marker to evaluate the EMT status of CTCs [114]. The mechanisms of Snail 1, Slug and ZEB 1 and their role in EMT are similar to that of Twist [22]. The PI3K/AKT/m TOR pathway is an important regulator of the cell cycle and related to proliferation, cancer and EMT [115]. As the central elements in this pathway, PI3K and Akt have already been used in some studies as mesenchymal markers of CTCs [102, 116].

The detection of these EMT markers in CTCs would help to evaluate their EMT status and distinguish CTC subpopulations.

### The expression of EMT markers in CTCs

In recent years, an increasing number of studies have attempted to detect EMT markers in CTCs. For example, Kasimir-Bauer, S et al. studied three EMT markers (Twist 1, Akt2, and PI3Kα) and one stem-cell marker (ALDH 1) in CTCs from 502 patients with primary breast cancer and detected at least one of the EMT markers and ALDH 1 in up to 72% and 46% of the CTC-positive group, respectively [102]. Kallergi, G et al. reported that over 80% of patients with both early and metastatic breast cancer harbored CTCs expressing phospho-Akt and phsopho-PI3K [117]. Moreover, Aktas, B et al. detected EMT markers and stem cell markers in 62% and 69% of the CTC-positive patients with metastatic breast cancer, respectively [103], and Li, YM et al. showed that the 80.4% and 84.8% of CTC isolated from patients with hepatocellular carcinoma express vimentin and Twist, respectively [118]. Similar findings have been reported in colorectal cancer, pancreatic cancer and prostate cancer [119–122].

Based on these data, we can conclude that EMT and stem cell markers are frequently overexpressed in CTCs, irrespective of cancer type. Remarkably, in addition to detecting EMT markers and ALDH 1 in the CTC-positive group, Kasimir-Bauer detected these respective markers in 18% and 5% of the CTC-negative group [102]. Aktas, B also reported the expression of EMT markers and stem cell markers in the CTC-negative group [103]. Gradilone, A et al. reported similar findings [123]. The three studies all relied on EpCAM-based capturing methods, i.e., AdnaTest and CELLection, to detect CTC and consequently may have missed CTC subpopulations that had undergone EMT, indicating that epithelial marker-based (such as EpCAM) detection methods may be unsuitable for detecting EMT CTCs. This notion is supported by several studies showing that combining EMT markers with epithelial markers increases the CTC detection rate compared with the AdnaTest approach [124].

### The expression of EMT markers in CTC correlates with tumor stage and metastasis

Kallergi, G et al. investigated the expression of the EMT markers Twist 1 and vimentin in CTCs from 25 patients with metastatic and 25 patients with early breast cancer and identified Twist 1(+) and vimentin(+) CTCs in 73% and 77% of patients with early breast cancer, respectively, whereas CTCs from all patients with metastatic breast cancer were positive for these markers [125]. Papadaki, M.A et al. also detected high ALDH1 expression (ALDH 1^high^) and nuclear Twist (Twist^nuc^) CTCs in 80% of patients with metastatic breast cancer but only 30.8% of patients with early breast cancer [126]. Moreover, Gradilone, A et al. evaluated the expression of vimentin, fibronectin and ALDH 1 in CTCs from 92 female patients with breast cancer and found that 91% (10/11) of the triple-positive samples (ALDH1/vimentin/ fibronectin) were isolated from patients with stage IV disease, and the expression of ALDH 1 by CTCs significantly correlated with the stage of disease [123]. Li, Y.M and colleges confirmed the correlation between Twist/vimentin expression and portal vein tumor thrombus or cancer stage in hepatocellular carcinoma, but they failed to find similar positive results with EMT-TFs, ZEB1, ZEB2 and Snail [118]. Nevertheless, Alonso-Alconada L et al. found that ZEB1 expression correlated with lymph node metastasis in endometrial cancer [127]. Similarly, Kulemann B reported this relationship in pancreatic ductal adenocarcinoma (PDCA) [120]. Although several meaningful findings have been made in several cancer types, Kasimir-Bauer, S et al. did not identify a correlation between EMT marker expression and cancer stage in their study [102].

The above studies suggest that the expression of EMT and stem cell markers in CTC may indicate a later stage and more aggressive disease but the clinical relevance of these findings needs to be further evaluated in more prospective trials.

### The expression of EMT markers in CTC correlates with therapeutic response

Aktas, B and colleges evaluated the expression of EMT and stem cell markers in CTCs isolated from patients with breast cancer during follow-up and detected EMT marker and ALDH1 expression in only 10% and 5% of responders, respectively, whereas these percentages were much higher in non-responders (62% and 44%, respectively) [103]. Mego, M et al. also reported that EMT marker positivity correlated with a lack of response to therapy and relapse [129]. Satelli, A et al. defined a threshold of 5 EMT CTCs to reflect therapeutic response in patients with metastatic colon cancer who received adjuvant chemotherapy after surgery: the patients with < 5 EMT CTCs responded to therapy, whereas patients with ≥ 5 EMT CTCs tended to experience disease progression [134]. Moreover, Chang K et al. found that stem cell marker expression indicated a worse response rate to chemotherapy in metastatic castration-resistant prostate cancer (mCRPC), but similar correlations with EMT markers were not identified [128]. Similarly, Polioudaki H et al. also failed to identify a correlation between EMT marker expression and therapeutic response in metastatic breast cancer [130].

These studies suggest that EMT and stem cell markers may be negatively correlated with therapeutic response, and the detection of EMT and stem cell markers in CTC may consequently be more convenient and faster than conventional methods to assess therapy response and help doctors manage patients with cancer better. However, the utility of EMT and stem cell markers in assessing therapeutic response needs to be fully confirmed and strictly standardized prior to clinical application.

### The expression of EMT markers in CTC correlates with prognosis

Lindsay, CR and colleges found that overall survival (OS) was significantly longer in patients with mCRPC whose CTCs were positive for the EMT marker vimentin than in patients whose CTCs lacked vimentin expression (453 days vs 305 days) [121]. Furthermore, Polioudaki H et al. detected keratin expression in CTCs from patients with metastatic breast cancer and found that low keratin expression correlated with worse OS [130]. Likewise, Ning, Y et al. showed that progression-free survival (PFS) (3.0 vs 7.7 months) and OS (10.0 vs 26.8 + months) were significantly shorter in patients with metastatic colorectal cancer with CTCs expressing ALDH1, PI3α and/or Akt-2 [119]. Mego, M also identified a correlation between EMT marker positivity and prognosis in patients with metastatic breast cancer [129]. Although an increasing number of studies have indicated that EMT marker-positive CTCs are valuable for prognosis, some studies failed to obtain similar results. Chang K et al. found that the expression of stem cell markers indicated poor prognosis in mCRPC patients, but EMT marker expression failed to show any prognostic value [128]. Moreover, Kulemann B's study of pancreatic ductal adenocarcinoma indicated a prognostic value for the KRAS mutation in CTCs but not the EMT marker ZEB1 [120]. Kasimir-Bauer, S et al. also failed to correlate the expression of EMT markers in CTCs with prognosis [102].

These studies indicate that the expression of EMT and stem cell markers may correlate with poor prognosis, but their prognostic value has to be further evaluated in additional clinical trials.

### Classifying CTCs based on the expression of EMT markers

EMT is likely to result in many hybrid phenotypes of CTCs that possess both epithelial and mesenchymal features because the extent of EMT may differ among CTCs [88]. Thus, in addition to the information provided by the detection of EMT markers in CTCs on cancer stage, treatment response and prognosis, some studies have attempted to classify CTCs based on EMT status to research the potential value of CTC subpopulations and further explore the relationship between the EMT in CTCs and cancer progression.

After detecting the expression levels of keratin and vimentin in breast cell lines and patients with breast cancer, Polioudaki H and colleges introduced a novel parameter, the vimentin/keratin ratio (Vim/K ratio), to reflect the EMT status of CTCs: a low Vim/K ratio suggested an epithelial phenotype, whereas a high Vim/K ratio indicated a mesenchymal phenotype in CTCs. Based on the Vim/K ratio of CTCs, they classified the CTCs into epithelial, biophenptypic and mesenchymal CTCs [130]. Moreover, Nel, I et al. counted the CK-positive, vimentin-positive and N-cadherin-positive CTCs and calculated the vimentin+ cells/CK+ cell ratio and N-cadherin+ cells/CK+ cell ratio in patients with hepatocellular carcinoma. They found that a change in the ratio of epithelial to mesenchymal CTCs was associated with a longer median time to progression (TTP) (1 vs 15 months), and the N-cadherin/CK ratio significantly correlated with cirrhosis [135]. Furthermore, Liu, YK et al. evaluated the expression of several EMT markers, including CK8/18/19, EpCAM, vimentin and Twist, in the CTCs of patients with hepatocellular carcinoma patients and classified CTCs into epithelial, biophenptypic and mesenchymal phenotypes based on the expression of EMT markers, which is the approach used by most studies to classify CTCs. Liu and colleges further studied the potential values of different CTC phenotypes and found that the number of epithelial CTCs was related to tumor size; biophenotypic CTCs were related to tumor number and mesenchymal CTCs were associated with metastasis [131]. Similarly, Zhao, R et al. analyzed the CTCs of patients with colorectal cancer and found that both biophenotypic and mesenchymal CTCs, but not epithelial CTCs, correlated with a late clinical stage, lymph node metastasis and distant metastasis, suggesting that CTCs with mesenchymal properties denote more aggressive disease and metastatic potential [132]. Li, TT classified CTCs from patients with gastric cancer into five types based on a similar principle and observed that the proportion of mesenchymal CTCs in the post-treatment blood specimens increased in a patient who experienced disease progression [133]. Accordingly, Yu, M et al. followed the EMT status of CTCs in patients with breast cancer and found that the EMT status of CTCs dynamically changed in response to therapy and disease progression [136], suggesting that the EMT status of CTCs may be used to monitor therapy response and cancer progression [133, 136].

## CONCLUSIONS AND PROSPECTIVE

EMT promotes the generation of circulating tumor cells (CTCs) in epithelial cancers by increasing tumor cell invasiveness and motility to promote tumor cell intravasation and facilitate tumor cell survival in the peripheral system. Although the contribution of EMT to tumor cell invasiveness has been confirmed, its role in metastasis remains debated. Two metastatic models involving EMT have been proposed: the classic EMT/MET model, in which EMT comprises the core of the metastatic cascade, and the collective migration model, in which EMT assists the early stage of the metastatic cascade, as discussed above. The metastatic cascade is a complex process, and its individual steps need to be elucidated before we can determine which of these models is correct. Based on limited current evidence, we herein hypothesized that the two models mentioned above may co-exist and synergistically facilitate the metastatic cascade; tumor cells may switch between mechanisms to maximize the rate of metastasis. However, several areas remain to be explored: the validity of the co-existence of the two models, the circumstances inducing cells to switch between motility patterns, and the routes of metastasis favored by each model. To this end, better-designed assays that can distinguish the two models and truly delineate each step of the metastatic cascade are needed. In conclusion, the extent to which EMT is necessary and the model responsible for the metastatic cascade remain to be determined.

Liquid biopsy is undoubtedly superior to conventional methods for dynamically monitoring cancer status, and the detection of CTCs is likely to gain popularity in the clinic. As a supplement to CTC enumeration, evaluating the expression of EMT and stem cell markers in CTCs may provide information about the tumor stage, metastasis, therapeutic response and prognosis, and using EMT markers to classify CTCs can elucidate CTC heterogeneity. However, standard and optimized approaches are lacking and studies of CTCs generally suffer from small sample sizes. Therefore, larger well-designed clinical trials are needed to further illuminate the potential values of EMT markers in CTCs.

## SUPPLEMENTARY MATERIALS TABLE


