# Supplementary material for: Epithelial-to-mesenchymal transition, circulating tumor cells and cancer metastasis: Mechanisms and clinical applications
Source: Oncotarget. 2017 May 26;8(46):81558–71. doi: 10.18632/oncotarget.18277 (PMC5655309; doi:10.18632/oncotarget.18277)
Supplement: Supplementary file 1 [file oncotarget-08-81558-s001.pdf]

Epithelial-to-mesenchymal transition, circulating tumor cells and cancer metastasis: Mechanisms and clinical applications

SUPPLEMENTARY MATERIALS

Supplementary Table 1: Studies on the expression and potential clinical relevance of EMT markers in CTCs of different types of cancer. See Supplementary_Table_1

REFERENCES

137. Raimondi C, Gradilone A, Naso G, Vincenzi B, Petracca A, Nicolazzo C, Palazzo A, Saltarelli R, Spremberg F, Cortesi E, Gazzaniga P. Epithelial-mesenchymal transition and stemness features in circulating tumor cells from breast cancer patients. Breast Cancer Res Treat. 2011; 130:449–455.
